# Serum lactate and mean arterial pressure thresholds in patients with cirrhosis and septic shock

**DOI:** 10.1097/HC9.0000000000000353

**Published:** 2024-01-05

**Authors:** Thomas N. Smith, Chansong Choi, Puru Rattan, Laura Piccolo Serafim, Blake A. Kassmeyer, Ryan J. Lennon, Ognjen Gajic, Jody C. Olson, Patrick S. Kamath, Alice Gallo De Moraes, Douglas A. Simonetto

**Affiliations:** 1 Department of Internal Medicine, Mayo Clinic Rochester, Rochester, Minnesota, USA; 2 Division of Gastroenterology and Hepatology, Mayo Clinic Rochester, Rochester, Minnesota, USA; 3 Division of Pulmonary and Critical Care Medicine, Mayo Clinic Rochester, Rochester, Minnesota, USA; 4 Division of Biomedical Statistics and Informatics, Mayo Clinic Rochester, Rochester, Minnesota, USA

## Abstract

**Background:**

The Sepsis-3 guidelines have incorporated serum lactate levels of >2 mmol/L in septic shock definition to account for higher observed mortality. Further evidence is needed to support this threshold in cirrhosis, as well as target mean arterial pressure (MAP) during resuscitation.

**Methods:**

This observational cohort study investigated the association between initial serum lactate and resuscitation MAP levels on in-hospital mortality in patients with and without cirrhosis. Patients admitted to the intensive care unit for the treatment of septic shock between 2006 and 2021 in a quaternary academic center were included. Patients with cirrhosis documented on imaging and International Classification of Disease codes (n=595) were compared to patients without cirrhosis (n=575). The association of intensive care unit admission lactate levels and median 2-hour MAP with in-hospital mortality and the need for continuous renal replacement therapy was assessed. The association between median 24-hour MAP and in-hospital mortality was analyzed post hoc.

**Results:**

Within the cirrhosis group, admission lactate levels of 2–4 and >4 mmol/L were associated with increased in-hospital mortality compared to lactate <2 mmol/L [adjusted odds ratio (aOR): 1.69, CI: 1.03–2.81, aOR: 4.02, CI: 2.53–6.52]. Median 24-hour MAP 60–65 and <60 mm Hg were also associated with increased in-hospital mortality compared with MAP >65 mm Hg (aOR: 2.84, CI: 1.64–4.92 and aOR: 7.34, CI: 3.17–18.76). In the noncirrhosis group, associations with in-hospital mortality were weaker for lactate 2–4 and >4 mmol/L (aOR: 1.32, CI: 0.77–2.27 and aOR: 2.25, CI: 1.40–3.67) and median 24-hour MAP 60–65 and <60 mm Hg (aOR: 1.70, CI: 0.65–4.14 and aOR: 4.41, CI: 0.79–29.38).

**Conclusions:**

These findings support utilizing lactate >2 mmol/L in the definition of septic shock, as well as a target MAP of >65 mm Hg during resuscitation in patients with cirrhosis.

## Graphical abstract

**Figure:**
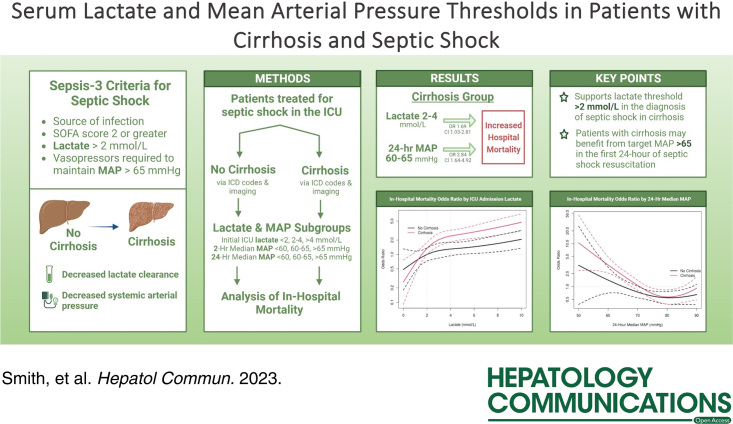

## INTRODUCTION

Sepsis and septic shock are syndromes of infection-induced pathobiological dysfunction with a significant disease burden at both the national and global levels. The criteria for the identification of septic shock were revised in 2016 to better reflect the known pathophysiology, emphasizing cellular metabolic dysfunction in addition to acute circulatory failure.^1^ A lower serum lactate cutoff was selected because of the increased risk-adjusted hospital mortality in those who also experienced hypotension requiring pressors. Since this change, it has been demonstrated that elevated lactate levels have prognostic value independent of pressor requirement in patients with septic shock, making it a sensitive marker of cellular metabolism during shock resuscitation.^2^

Patients with advanced cirrhosis have an increased risk of infection due to their impaired immune system, with up to one-third developing sepsis during hospitalization.^3,4^ Mortality in patients with cirrhosis and septic shock in the intensive care unit (ICU) setting remains as high as 65% despite improvements over the last decade.^5^ Infection alone confers up to a four-fold increase in mortality in this population.^6^ Independent of infection and sepsis, patients with cirrhosis experience baseline derangements in hemodynamics, cellular metabolism, and lactate clearance, which may confound detection and risk stratification of sepsis and septic shock.^7^ Of note, the sequential organ failure assessment (SOFA) score, part of the Sepsis-3 criteria for sepsis, has been demonstrated to predict in-hospital mortality more effectively in patients with cirrhosis than the previously utilized systemic inflammatory response syndrome criteria.^8^

Typical changes in cirrhosis include hyperdynamic circulation, abnormal blood volume distribution, and neurohumoral dysregulation.^9^ Acute on chronic liver failure, a diagnosis associated with high systemic inflammation, is commonly accompanied by distributive circulatory failure resulting in signs of impaired tissue perfusion.^10^ These derangements in typical physiology may complicate the diagnosis of septic shock in these patients, and it is worthwhile to further clarify the appropriate goals for hemodynamic support to improve outcomes.

For the general population, a mean arterial pressure (MAP) target of 65–70 mm Hg has been suggested during septic shock resuscitation.^11^ The accepted MAP target during shock resuscitation in patients with cirrhosis remains controversial due to the lack of supporting evidence in this population. A lower threshold has been previously suggested based on expert opinion due to the baseline circulatory derangements observed in cirrhosis,^10^ although discussion regarding appropriate MAP targets is ongoing. While these patients have increased incidence and mortality associated with sepsis, the prognostic value of serum lactate and MAP for risk-adjusted hospital mortality has not been clearly demonstrated in this population.

The aim of our study was to investigate the lactate cutoff for septic shock definition and target MAP during resuscitation, their association with in-hospital mortality, and the need for continuous renal replacement therapy (CRRT) in a cohort of patients with and without cirrhosis.

## METHODS

### Study design

This observational cohort study evaluated adults admitted to ICUs at Mayo Clinic for the treatment of septic shock between January 2006 and December 2021. Sepsis was defined as a SOFA score of 2 or greater with positive cultures (blood, ascites, urine) or chest imaging concerning bacterial pneumonia (new consolidation, air bronchograms, or infiltrates on x-ray or CT). Septic shock was defined as sepsis plus vasopressor use within the first 6 hours of ICU admission and lactate value obtained on ICU admission. These encounters were identified using a validated ICU Datamart.^12^ Cases of cirrhosis were determined using International Classification of Disease (ICD) codes and confirmed using a natural language process algorithm (Supplemental Table S1, http://links.lww.com/HC9/A712) to identify features of cirrhosis and portal hypertension on imaging studies (ultrasound, CT, or MRI). Cases without cirrhosis were determined by the absence of ICD codes for cirrhosis and confirmed by ruling out features of cirrhosis and portal hypertension on imaging studies using this natural language process algorithm. Readmissions were excluded, and the cases of cirrhosis were compared with patients without cirrhosis, as determined by the absence of any cirrhosis-related ICD codes. Subgroups were created based on ICU admission lactate levels (<2, 2–4, >4 mmol/L) and median 2-hour MAP (<60, 60–65, >65 mm Hg), and in-hospital mortality and need for CRRT were compared. Post hoc analysis evaluated median 24-hour MAP and in-hospital mortality and the need for CRRT in the cirrhosis and noncirrhosis groups. The exclusion criteria included cardiogenic cirrhosis, prior liver transplant, metastatic malignancies, current chemotherapy, age <18 years, lack of research authorization, and admission for surgical management. All research was conducted in accordance with both the Declarations of Helsinki and Istanbul, approved by the appropriate institutional review committee, and written consent was given by all subjects.

### Data collection

Data collected for these patients included demographic characteristics, comorbid medical diagnoses, Acute Physiology and Chronic Health Evaluation (APACHE) III, SOFA scores on day 1 of admission, hemodynamic measurements, laboratory data on ICU admission, bacterial culture data, chest imaging information, vasopressor use during ICU admission, need for renal replacement therapy, and outcomes. The Model for End-Stage Liver Disease scores were calculated using this information, as outlined in the literature.^13,14^

### Data analysis

Numeric variables were summarized using the mean, SD, median, and first and third quartiles. Categorical variables were described using counts and frequencies. Unadjusted outcome comparisons by lactate and MAP level groups were conducted using the Armitage Trend Test. Patient characteristics and demographic comparison tests between patients with and without cirrhosis were conducted using a 2-sample *t* test for numeric variables and chi-square test for categorical variables. Plots using natural cubic splines for lactate and MAP were estimated using patients without cirrhosis with 2 mmol/L and 65 mm Hg as reference, respectively. The association of lactate levels at ICU admission and median 2-hour MAP with in-hospital mortality and the need for CRRT was evaluated using multiple logistic regression. Firth bias-reduction method was applied to allow for estimation in the presence of numerical separation.^15^ All models were adjusted for age and sex. The primary analysis models used grouped categories for lactate levels and MAP levels, with associations reported as odds ratios and 95% CIs. Secondary models used natural cubic spline terms for lactate and MAP levels. A similar post hoc analysis was performed regarding median 24-hour MAP on in-hospital mortality and the need for CRRT during hospitalization. Analyses were conducted using R 4.1.2 (R Foundation).

## RESULTS

A total of 595 patients with cirrhosis and 575 without cirrhosis who were admitted to the ICU for the treatment of septic shock were included in the study. Demographic information, illness severity indices, and medical comorbidities were compared between groups (Table 1).

**TABLE 1:** Group comparison of demographics, illness indices, and medical comorbidities

	No cirrhosis (N=575)	Cirrhosis (N=595)	*p*
Age, y			0.077
Median (Q1, Q3)	62.6 (52.7, 71.4)	60.5 (51.6, 68.9)	
Sex, n (%)			0.440
Female	225 (39.1)	246 (41.3)	
Male	350 (60.9)	349 (58.7)	
BMI			0.050
Median (Q1, Q3)	29.4 (24.3, 34.5)	27.7 (23.5, 33.4)	
Hospital LOS			0.763
Median (Q1, Q3)	12.9 (7.3, 26.7)	13.5 (7.2, 25.4)	
ICU LOS			0.594
Median (Q1, Q3)	3.8 (2.0, 7.7)	3.9 (1.9, 8.3)	
APACHE III score (24 h)			<0.001
Median (Q1, Q3)	88.0 (69.0, 113.0)	98.0 (78.0, 121.5)	
SOFA score (24 h)			<0.001
Median (Q1, Q3)	10.0 (7.0, 12.0)	12.0 (9.0, 14.0)	
MELD score (24 h)			
Median (Q1, Q3)	NA	23.0 (17.5, 30.4)	NA
Dialysis during hospitalization, n (%)			<0.001
No	438 (76.2)	397 (66.7)	
Yes	137 (23.8)	198 (33.3)	
Invasive ventilation use, n (%)			0.751
No	155 (27.1)	155 (26.2)	
Yes	418 (72.9)	436 (73.8)	
Comorbidities, n (%)			
Chronic kidney disease	185 (32.2)	275 (46.2)	<0.001
Diabetes mellitus	203 (35.3)	247 (41.5)	0.029
Chronic obstructive pulmonary disease	94 (16.3)	110 (18.5)	0.335
Coronary artery disease	183 (31.8)	162 (27.2)	0.085

Abbreviations: APACHE, Acute Physiology and Chronic Health Evaluation; BMI, Body Mass Index; ICU, intensive care unit; LOS, length of stay; MELD, Model for End-Stage Liver Disease; NA, not available; SOFA, sequential organ failure assessment.

### Admission lactate threshold

Although higher mortality was observed in patients with cirrhosis in both the lactate 2–4 mmol/L (cirrhosis, 27.0%; noncirrhosis, 20.9%) and >4 mmol/L subgroups (cirrhosis, 45.7%; noncirrhosis, 30.0%), there was no significant evidence (*p*=0.24) of an interaction between cirrhosis and lactate levels (Table 2). After adjusting for age and sex, patients with cirrhosis still had an elevated risk of in-hospital mortality at the lower lactate threshold [adjusted odds ratio (aOR): 1.69, 95% CI: 1.03–2.81 for lactate 2–4; aOR: 4.02, 95% CI: 2.53–6.52 for lactate >4; Table 3]. These associations were not significantly different from those in patients without cirrhosis (interaction, *p*=0.22). A similar interaction test for cirrhosis and admission lactate level on the need for CRRT was also not significant (*p*=0.19).

**TABLE 2:** Risk of in-hospital mortality by lactate and MAP groups, within patients with and without cirrhosis

	No cirrhosis			Cirrhosis		
	N	In-hospital death, % (n)	*p*	N	In-hospital death, % (n)	*p*
Admission lactate, mmol/L						
<2	200	16.0 (32)	0.003	174	17.8 (31)	<0.001
2–4	158	20.9 (33)		200	27.0 (54)	
>4	217	30.0 (65)		221	45.7 (101)	
Median 2-h MAP, mm Hg						
>65	429	22.8 (98)	0.32	379	29.6 (112)	0.44
60–65	81	17.3 (14)		92	32.6 (30)	
<60	65	27.7% (18)		124	35.5% (44)	
Median 24-h MAP, mm Hg						
>65	549	21.9 (120)	0.062	509	26.7 (136)	<0.001
60–65	21	33.3 (7)		60	51.7 (31)	
<60	5	60.0 (5)		26	73.1 (19)	

Abbreviation: MAP, mean arterial pressure.

**TABLE 3:** Adjusted^a^ ORs and 95% CIs for lactate and MAP groups and comparison between patients with and without cirrhosis, for both in-hospital mortality and CRRT

	In-hospital mortality			CRRT		
	OR (CI)			OR (CI)		
	No cirrhosis	Cirrhosis	*p* ^b^	No cirrhosis	Cirrhosis	*p* ^b^
Admission lactate, mmol/L			0.22			0.19
<2	1.00 (reference)	1.00 (reference)^c^		1.00 (reference)	1.00 (reference)^c^	
2–4	1.32 (0.77, 2.27)	1.69 (1.03, 2.81)		0.97 (0.48, 1.95)	1.43 (0.82, 2.55)	
>4	2.25 (1.40, 3.67)	4.02 (2.53, 6.52)		3.44 (2.03, 6.05)	2.53 (1.52, 4.34)	
Median 2-h MAP, mm Hg			0.50			0.62
>65	1.00 (reference)	1.00 (reference)^c^		1.00 (reference)	1.00 (reference)^c^	
60–65	0.72 (0.38, 1.30)	1.14 (0.69, 1.86)		1.33 (0.71, 2.39)	0.88 (0.48, 1.56)	
<60	1.30 (0.71, 2.32)	1.33 (0.86, 2.04)		1.37 (0.69, 2.56)	1.31 (0.80, 2.10)	
Median 24-h MAP, mm Hg			0.57			0.74
>65	1.00 (reference)	1.00 (reference)^c^		1.00 (reference)	1.00 (reference)^c^	
60–65	1.70 (0.65, 4.14)	2.84 (1.64, 4.92)		1.73 (0.58, 4.43)	1.41 (0.74, 2.57)	
<60	4.41 (0.79, 29.4)	7.34 (3.17, 18.8)		0.50 (<.01, 4.52)	1.30 (0.48, 3.08)	

a All estimates are adjusted for age and gender. All models included appropriate main effects for the interaction analysis.

b *p* values for the test of interaction between the presence of cirrhosis and the lactate or MAP group.

c ORs within the table represent the increased risk of the endpoint within the no cirrhosis and cirrhosis groups.

The main effect of cirrhosis was also included in all models. Due to the interaction term, this effect represents only the cirrhosis effect in those with low lactate or high MAP values. For in-hospital mortality, the cirrhosis effect was estimated as OR: 1.18 (95% CI: 0.68, 2.03) in the lactate model, OR: 1.49 (95% CI: 1.08, 2.05) in the 2-hour MAP model, and OR: 1.37 (95% CI: 1.03, 1.82) in the 24-hour MAP model. For CRRT, the cirrhosis effect was estimated as OR: 1.35 (95% CI: 0.72, 2.56) in the lactate model, OR: 1.31 (95% CI: 0.91, 1.88) in the 2-hour MAP model, and OR: 1.21 (95% CI: 0.89, 1.66) in the 24-hour MAP model.

Abbreviations: CRRT, continuous renal replacement therapy; MAP, mean arterial pressure.

**FIGURE 1:**
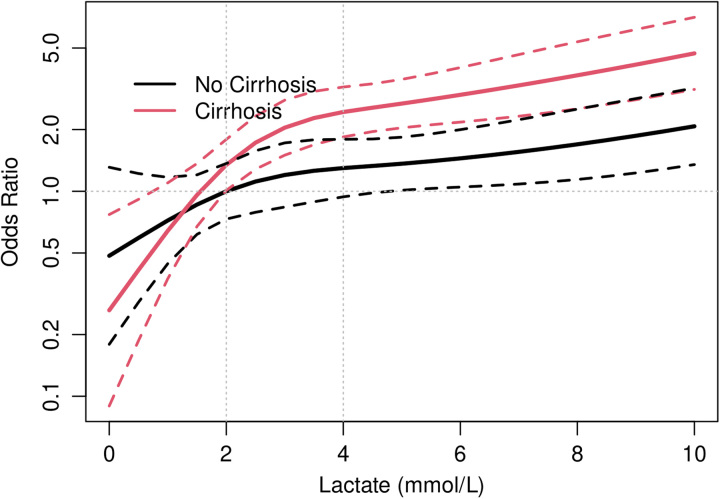
In-hospital mortality OR by ICU admission lactate. Plot of in-hospital mortality OR (adjusted for age and sex) by ICU admission lactate for both cirrhosis and noncirrhosis groups. Abbreviation: ICU, intensive care unit.

The adjusted association of initial ICU lactate level with in-hospital mortality was also analyzed on a continuous scale, with separate associations for patients with and without cirrhosis. Figure 1 reflects these associations relative to a patient without cirrhosis and an initial lactate level of 2 mmol/L, demonstrating a relative plateauing of mortality risk after admission lactate levels increase past 2 mmol/L for both groups.

An initial lactate level >4 mmol/L was also associated with an increased risk of CRRT in both the cirrhosis (aOR: 2.53, CI: 1.52–4.34) and noncirrhosis (aOR: 3.44, CI: 2.03–6.05) groups. This association was not significant in initial lactate 2–4 mmol/L for the cirrhosis (aOR: 1.43, CI: 0.82–2.55) or noncirrhosis (aOR: 0.97, CI: 0.48–1.95) groups. There was no significant interaction (*p*=0.19) between cirrhosis and lactate levels.

### Median 2-hour MAP

**FIGURE 2:**
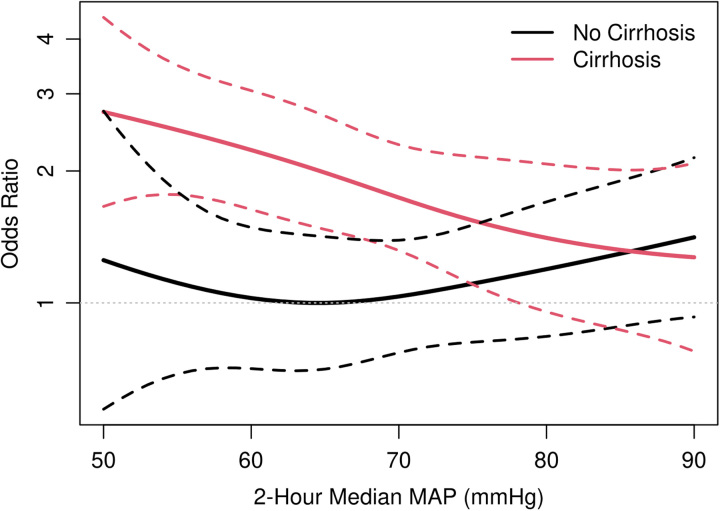
In-hospital mortality OR by median 2-hour MAP. Plot of in-hospital mortality OR (adjusted for age and sex) by median 2-hour MAP for both cirrhosis and noncirrhosis groups. Abbreviation: MAP, mean arterial pressure.

An age-adjusted and sex-adjusted model for in-hospital mortality did not find a significant interaction (*p*=0.50) between median 2-hour MAP and cirrhosis (Table 3). Within the cirrhosis group, median 2-hour MAP 60–65 (aOR: 1.14, CI: 0.69–1.86) and <60 mm Hg (aOR: 1.33, CI: 0.86–2.04) were not found to have increased mortality when compared with MAP >65 mm Hg. Similarly, within the noncirrhosis group, median 2-hour MAP of 60–65 (aOR: 0.72, CI: 0.38–1.30) and <60 mm Hg (aOR: 1.30, CI: 0.71–2.32) were not found to have increased mortality when compared with MAP >65. Figure 2 demonstrates the continuous association between 2-hour MAP and the risk of in-hospital mortality in patients with and without cirrhosis. Similarly, no significant interaction (*p*=0.62) was found between cirrhosis and 2-hour median MAP on the need for CRRT, nor were MAP groups significantly associated with in-hospital mortality (Table 3).

### Median 24-hour MAP

**FIGURE 3:**
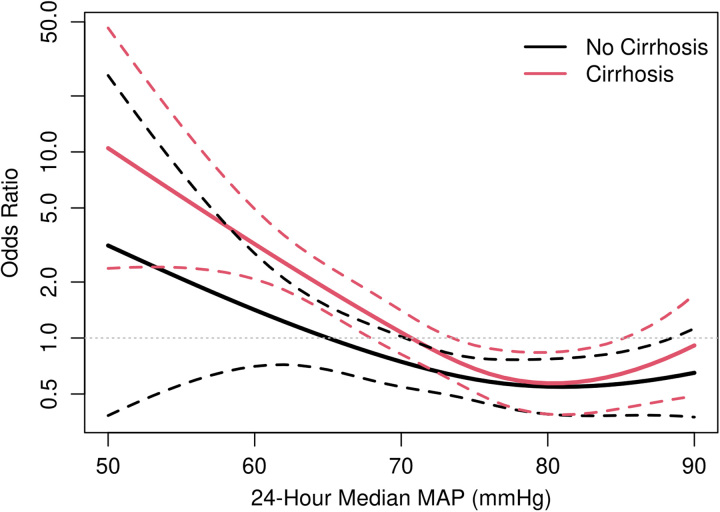
In-hospital mortality OR by median 24-hour MAP. Plot of in-hospital mortality OR (adjusted for age and sex) by median 24-hour MAP for both cirrhosis and noncirrhosis groups. Abbreviation: MAP, mean arterial pressure.

Given the neutral effect of MAP within the initial 2 hours of ICU admission in both groups with and without cirrhosis, a post hoc analysis was performed to evaluate the association of median MAP in the first 24 hours of ICU admission with hospital mortality. When comparing patients with and without cirrhosis, the overall interaction between median 24-hour MAP and cirrhosis on in-hospital mortality (*p*=0.57) and the need for CRRT (*p*=0.74) was not significant. In patients with cirrhosis, both median 24-hour MAP 60–65 (aOR: 2.84, CI: 1.64–4.92) and <60 mm Hg (OR: 7.34, CI: 3.17–18.76) were significantly associated with increased in-hospital mortality compared with MAP >65 mm Hg. In patients without cirrhosis, most (95%) had MAP >65 mm Hg, which limited our statistical power to detect differences at lower MAP values. Figure 3 presents the association between the 24-hour median MAP and the risk of in-hospital mortality. No significant associations or interactions were observed between 24-hour MAP and the risk of CRRT (Table 3). Total vasopressor use was calculated for both cirrhosis and noncirrhosis groups, and there was no significant difference seen in norepinephrine equivalents administered (Supplemental Table S2, http://links.lww.com/HC9/A712).

## DISCUSSION

Patients with cirrhosis are frequently underrepresented in major studies guiding the current management of septic shock, and the impact of physiological changes observed in cirrhosis on the appropriate management of septic shock remains unclear.^16^ This observational study of patients treated for septic shock found that the currently accepted thresholds for initial serum lactate and resuscitation MAP targets were consistent with observed in-hospital mortality in patients with cirrhosis.

After adjusting for age and sex, our models did not demonstrate significant interactions between the diagnosis of cirrhosis and initial serum lactate, median 2-hour MAP, or median 24-hour MAP, and their association with in-hospital mortality or the need for CRRT during hospitalization. Overall, these findings suggest that the relative effects of serum lactate and MAP measures may be similar in patients with and without cirrhosis in the setting of septic shock. As expected, patients in the cirrhosis group were more critically ill, as demonstrated by the comparison of APACHE III and SOFA scores (Table 1), which explains the difference in overall in-hospital mortality seen between the cirrhosis and noncirrhosis groups.

Although lactate is often elevated in patients with cirrhosis due to decreased liver gluconeogenesis and clearance, prior studies have shown that it remains an independent predictor of mortality in patients with cirrhosis and critical illness.^17–19^ Our study provides additional evidence for the clinical significance of elevated initial serum lactate levels in patients with cirrhosis and septic shock. While a recent retrospective study did not find initial lactate to be an independent predictor of in-hospital mortality in cirrhosis,^20^ our findings demonstrate that initial lactate elevation even between 2 and 4 mmol/L is associated with increased in-hospital mortality. The analysis of the lactate association with in-hospital mortality as a continuous variable (Figure 1) demonstrated similar trends in both the cirrhosis and noncirrhosis groups across this lactate threshold. Together, these findings support the use of the Sepsis-3 lactate cutoff of 2 mmol/L for the diagnosis of septic shock in patients with cirrhosis.

It has been suggested that dynamic measures of serum lactate, such as lactate clearance or time to normalization, maybe other important prognostic factors for patients with cirrhosis and shock, and previous studies have demonstrated an association between liver disease and delayed lactate clearance during septic shock resuscitation.^21^ Based on variable time intervals between resuscitation lactate values as well as missing lactate data, we were unable to effectively calculate lactate clearance for analysis in this study. However, this remains an area of high interest and further studies are needed to evaluate the effect of lactate clearance on mortality in septic shock in patients with cirrhosis.

The target MAP during septic shock resuscitation in patients with cirrhosis remains controversial. A recent randomized controlled trial by Maiwall et al^22^ evaluated high (80–85 mm Hg) versus low (60–65 mm Hg) MAP targets in patients with cirrhosis during septic shock resuscitation and found that a high MAP target was not associated with improved survival at 28 days. Of note, despite the 60–65 mm Hg target in the low MAP group, most patients were maintained above 65 mm Hg during the first 24 hours of resuscitation. Expert opinion has suggested that MAP targets <65 mm Hg during shock resuscitation might be acceptable in cirrhosis, which had led to variability in clinical practice, as demonstrated in our study, where a higher rate of patients with median MAP <65 mm Hg was seen in the cirrhosis versus noncirrhosis groups (36% vs. 25% at 2 h, 15% vs. 5% at 24 h). Our study’s finding of increased in-hospital mortality in patients with cirrhosis and median MAP 60–65 mm Hg over the first 24 hours of ICU management suggests that despite baseline alterations in hemodynamics, an MAP target of >65 mm Hg over this period might be beneficial. The linear plot of continuous 24-hour median MAP and OR of in-hospital mortality (Figure 3) demonstrated a similar slope for both cirrhosis and noncirrhosis groups at MAP 60–65, indicating that it may be appropriate to target MAP in cirrhosis similarly to the general population.

The systemic vasodilation and hyperdynamic circulation seen in patients with cirrhosis have been well described and likely impact the decreased response to crystalloid resuscitation seen in these patients compared to the general population.^23,24^ While these patients often tolerate lower MAP at baseline secondary to collateral blood flow, angiogenesis, and vascular remodeling,^25^ it is possible that these compensatory mechanisms are insufficient for organ perfusion during sepsis-related vasodilation and require higher MAP goals than at baseline. Further studies are needed to identify potential subgroups of patients with cirrhosis that may benefit from a low (60–65 mm Hg) MAP target during shock resuscitation, if any.

A recent study by Maiwall et al^22^ also suggested that higher MAP targets during shock resuscitation improved renal outcomes, such as renal recovery at 14 days. While lower MAP was not associated with increased CRRT need in our study, initial lactate >4 mmol/L was associated with higher rates of CRRT in both patients with and without cirrhosis. These findings highlight the potential role of initial ICU serum lactate levels in predicting the risk of CRRT requirement during hospitalization. Other markers of organ perfusion such as 24-hour urine output (UO) may also help us determine the clinical significance of arterial hypotension. While UO was previously thought to be an unreliable marker due to increased sodium avidity independent of renal dysfunction,^26^ more recent studies in the ICU setting have demonstrated that UO measurements can increase the sensitivity of acute kidney injury detection and predict increased mortality in patients with cirrhosis.^27,28^ This has resulted in some experts urging for its inclusion in acute kidney injury definitions within this population. UO remains an area of future interest as we refine our effective prognostication of mortality and the need for CRRT in patients with cirrhosis.

Because this retrospective study evaluates a 15-year period, analysis was performed on temporal trends in ICU care such as changes in resuscitation MAP levels and in-hospital mortality. While our institution did not make explicit changes in ICU practice regarding MAP targeting during septic shock resuscitation, we did find a gradual increase in median 24-hour MAP over time in both the cirrhosis and noncirrhosis groups (Supplemental Figure S1, http://links.lww.com/HC9/A712). In this plot of median 24-hour MAP as a function of admission year, we see MAP increase from 68.0 mm Hg (2006) to 75.3 mm Hg (2021) in the cirrhosis group and from 72.3 mm Hg (2006) to 76.9 mm Hg (2021) in the noncirrhosis group. Our in-hospital mortality analysis also included adjustments for admission year; however, our results did not significantly change for either group and were not included in our final analysis.

There are important limitations to this observational study that warrant further discussion. Because our hospital is a quaternary care center, a portion of our study population was transferred from outside facilities including ICUs. In some cases, bacterial culture data before antimicrobial administration, previous fluid and vasopressor resuscitation, and prior hemodynamic monitoring were unavailable. Of the ICU admissions included in this study, 11.4% came from our emergency department, which also includes many patients who were transferred from outside institutions but required initial evaluation or treatment in the emergency department. In all cases of septic shock, there is the potential for delay between the diagnosis and initial evaluation of patients and admission to the ICU. This creates difficulty in establishing uniform time points for the evaluation of both serum lactate levels and MAP. In this study, only initial admissions to the ICU for septic shock resuscitation were analyzed, which excludes potential readmissions for recurrent septic shock management. The diagnosis of sepsis in this study was based on culture positivity or chest imaging findings suggestive of bacterial pneumonia, which resulted in the exclusion of patients with culture-negative infections. Some estimates suggest that a large proportion of infections may remain culture-negative in this population.^29,30^ Although using such strict definitions of sepsis may have resulted in the selection of sicker patients, it increases the diagnostic certainty compared to prior studies. Another consideration is the possibility of culture positivity in the absence of clinically significant infection; however, the requirement of an SOFA score of 2 or greater as well as vasopressor administration within 6 hours of ICU admission limits the risk of confounding. The identification of patients with cirrhosis on electronic medical records also often represents a challenge given the variable accuracy of ICD codes.^31,32^ To increase the accuracy of cirrhosis diagnosis, we employed a natural language process model for the detection of imaging features of cirrhosis and portal hypertension. This approach may have led to the selection of patients with more advanced stages of cirrhosis. However, it arguably focused on the population of interest, that is, those more likely to experience hemodynamic changes at baseline.

Overall, the study findings support the continued use of serum lactate >2 mmol/L as a cutoff in the diagnosis of septic shock and an MAP target of 65 mm Hg or greater in the first 24 hours of septic shock resuscitation. Further studies are needed to validate our findings and to investigate the role of lactate clearance rate in outcome prediction in patients with cirrhosis.

## Supplementary Material

SUPPLEMENTARY MATERIAL
