# Supplementary material for: Serum lactate and mean arterial pressure thresholds in patients with cirrhosis and septic shock
Source: Hepatol Commun. 2024 Jan 5;8(1):e0353. doi: 10.1097/HC9.0000000000000353 (PMC10781124; doi:10.1097/HC9.0000000000000353)
Supplement: SUPPLEMENTARY MATERIAL [file hc9-8-e0353-s001.docx]

**Supplemental Table 1**: Imaging search terms and ICD codes used to determine cirrhosis diagnosis.

| Natural Language Processing (NLP) Imaging Search Terms |
| --- |
| Cirrhotic liver morphology  Atrophy of liver  Nodular contour  Portal hypertension  Patent paraumbilical vein  Portosystemic shunts  Varices  Coarsened hepatic echotexture  Consistent with cirrhosis  Cirrhosis  Cirrhotic |
| ICD-10 Codes |
| \| K70.30 \| Alcoholic cirrhosis of liver without ascites \| \| --- \| --- \| \| K70.31 \| Alcoholic cirrhosis of liver with ascites \| \| K70.40 \| Alcoholic hepatic failure without coma \| \| K70.41 \| Alcoholic hepatic failure with coma \| \| K74.4 \| Secondary biliary cirrhosis \| \| K74.5 \| Biliary cirrhosis, unspecified \| \| K74.60 \| Unspecified cirrhosis of liver \| \| K74.69 \| Other cirrhosis of liver \| |
| ICD-9 Codes |
| \| 571 \| Chronic liver disease and cirrhosis \| \| --- \| --- \| \| 571.2 \| Alcoholic cirrhosis of liver \| \| 571.5 \| Cirrhosis of liver without mention of alcohol \| \| 571.6 \| Biliary cirrhosis \| |

Natural Language Processing (NLP) and ICD codes used to determine cirrhosis diagnosis. Imaging search terms had to match an encounter involving CT, ultrasound, or MRI imaging.

**Supplemental Table 2:** Vasopressor Use During Resuscitation Time Intervals

| Norepinephrine Equivalents (mg) | No Cirrhosis | Cirrhosis | P-value |
| --- | --- | --- | --- |
| **24 hrs before ICU Admission** |  |  | 0.994 |
| Median (Q1, Q3) | 0.0 (0.0, 0.6) | 0.0 (0.0, 0.3) |  |
| Mean (SD) | 3.8 (16.7) | 3.8 (16.0) |  |
| **6 hrs after ICU Admission** |  |  | 0.998 |
| Median (Q1, Q3) | 2.4 (0.5, 20.5) | 2.6 (0.6, 17.9) |  |
| Mean (SD) | 11.8 (17.9) | 11.8 (17.6) |  |
| **12 hrs after ICU Admission** |  |  | 0.261 |
| Median (Q1, Q3) | 7.3 (1.6, 48.3) | 11.2 (1.9, 51.9) |  |
| Mean (SD) | 26.7 (35.2) | 29.1 (36.1) |  |
| **Total ICU during ICU Admission** |  |  | 0.811 |
| Median (Q1, Q3) | 17.7 (3.0, 118.4) | 21.1 (5.0, 165.6) |  |
| Mean (SD) | 171.3 (668.4) | 179.0 (402.3) |  |

Norepinephrine equivalents calculated using [ *NE = norepinephrine (mg) + epinephrine (mg) + phenylephrine (mg)/10 + dopamine (mg)/100 + vasopressin (units)*2.5 + angiotensin II (mg)*10]* via Goradia, et al (PMID: 33220576).

**Supplemental Figure 1:** Median 24-Hour MAP Trend by Admission Year


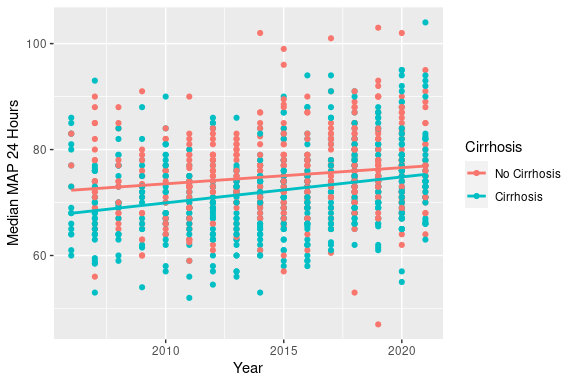


Plot of median 24-Hour MAP by admission year for both cirrhosis and non-cirrhosis groups.
